# Identification and characterization of two wheat Glycogen Synthase Kinase 3/ SHAGGY-like kinases

**DOI:** 10.1186/1471-2229-13-64

**Published:** 2013-04-18

**Authors:** Thomas Bittner, Sarah Campagne, Gunther Neuhaus, Stefan A Rensing, Christiane Fischer-Iglesias

**Affiliations:** 1 Cell Biology, Faculty of Biology, University of Freiburg, Schaenzlestr. 1, D-79104 Freiburg, Germany; 2 Faculty of Biology & BIOSS Centre for Biological Signalling Studies, University of Freiburg, Schaenzlestr. 1, D-79104 Freiburg, Germany; 3 Cell Biology, Faculty of Biology, Philipps-University Marburg, Karl-von-Frisch-Str. 8, D-35043 Marburg, Germany

**Keywords:** SHAGGY-like kinase, GSK-3-like kinase, *Poaceae*, Wheat, Homologs, Homoeologs, Phylogenetic analysis, Brassinosteroid signaling

## Abstract

**Background:**

Plant Glycogen Synthase Kinase 3/ SHAGGY-like kinases (GSKs) have been implicated in numerous biological processes ranging from embryonic, flower, stomata development to stress and wound responses. They are key regulators of brassinosteroid signaling and are also involved in the cross-talk between auxin and brassinosteroid pathways. In contrast to the human genome that contains two genes, plant GSKs are encoded by a multigene family. Little is known about Liliopsida resp*. Poaceae* in comparison to *Brassicaceae* GSKs. Here, we report the identification and structural characterization of two GSK homologs named *TaSK1* and *TaSK2* in the hexaploid wheat genome as well as a widespread phylogenetic analysis of land plant GSKs.

**Results:**

Genomic and cDNA sequence alignments as well as chromosome localization using nullisomic-tetrasomic lines provided strong evidence for three expressed gene copies located on homoeolog chromosomes for *TaSK1* as well as for *TaSK2*. Predicted proteins displayed a clear GSK signature. *In vitro* kinase assays showed that TaSK1 and TaSK2 possessed kinase activity. A phylogenetic analysis of land plant GSKs indicated that TaSK1 and TaSK2 belong to clade II of plant GSKs, the *Arabidopsis* members of which are all involved in Brassinosteroid signaling. Based on a single ancestral gene in the last common ancestor of all land plants, paralogs were acquired and retained through paleopolyploidization events, resulting in six to eight genes in angiosperms. More recent duplication events have increased the number up to ten in some lineages.

**Conclusions:**

To account for plant diversity in terms of functionality, morphology and development, attention has to be devoted to Liliopsida resp *Poaceae* GSKs in addition to *Arabidopsis* GSKs. In this study, molecular characterization, chromosome localization, kinase activity test and phylogenetic analysis (1) clarified the homologous/paralogous versus homoeologous status of *TaSK* sequences, (2) pointed out their affiliation to the GSK multigene family, (3) showed a functional kinase activity, (4) allowed a classification in clade II, members of which are involved in BR signaling and (5) allowed to gain information on acquisition and retention of GSK paralogs in angiosperms in the context of whole genome duplication events. Our results provide a framework to explore Liliopsida resp *Poaceae* GSKs functions in development.

## Background

Glycogen synthase kinase 3 (GSK-3) / SHAGGY kinase (SGG) are multifunctional non receptor serine/threonine kinases.

In humans and animals, GSK-3/SGG are key regulators of a broad range of signaling pathways and their dysregulation responsible for a number of diseases or developmental abnormalities, both aspects abundantly documented in the literature.

In humans, two enzymes named GSK-3β and GSK-3α, encoded by two genes, are involved in the regulation of glycogen metabolism [1], in the regulation of the cell cycle [2], in the stability of the cytoskeleton [3], in apoptosis [2,4], in the modulation of the activity of transcription factors such as c-Jun and c-Myc [2] and in a range of diseases including Alzheimer [4], and cancer [5].

SGG/GSK-3 are a master switch in the Wnt/Wingless(Wg) pathways and are involved in fundamental developmental processes in animals such as cell fate specification, pattern formation and body axis formation [2,6,7]. The regulation of SGG/GSK-3 represents a conserved strategy during evolution for establishing embryonic polarity of both invertebrate and vertebrates. In *Drosophila*, a pool of SGG isoenzymes encoded by a single gene is necessary to establish cell fate and polarity within embryonic segments [8] as well as for development of the nervous system [9]. Ventral injection of a catalytically inactive form of GSK-3β in *Xenopus laevis* embryos results in the induction of dorsal development and differentiation of ectopic supernumerary body axes indicating that GSK-3 regulates the dorso-ventral plan formation [10]. In *Hydra*, inhibition of activity of the HyGSK-3 confers characteristics of the head organizer to the body column resulting in the differentiation of ectopic heads and tentacles on the body column [11].

GSKs also exist in a number of plant species [7]. Although, investigations of plant GSK-3 started more recently, their roles appear also numerous. In contrast to animals, plant GSKs are encoded by a multigene family [12].

Most information available on their biological function and mechanism of action are provided by the study of BIN2 in *Arabidopsis.* BIN2/ASKη (Brassinosteroid insensitive2/ *Arabidopsis* Shaggy-related protein Kinase eta) and its two close relatives ASKiota and ASKdzeta, all three being members of clade II, are involved in brassinosteroid (BR) signaling [13,14]. Gain of function *bin2.1* mutation results in a dwarf phenotype resembling that of BR-deficient or BR signaling mutants [13,15]. BIN2 has a negative role in the BR signaling pathways [13]. The kinase phosphorylates the transcription factors Bri1-EMS-suppressor1 (BES1) and BrassinaZole-Resistant1(BZR1) in order to promote the protein degradation of BRZ1 [16], to affect the subcellular localization of BRZ1 and BES1 [17,18] and to affect both binding to target promoters and transcriptional activity of BES1 [14]. Upstream BR signaling is negatively regulating BIN2 protein level through proteasome mediated degradation [19] and inactivating BIN2 kinase activity by dephosphorylation of a conserved tyrosine residue [20]. Studies of the *ULTRACURVATA1* gene that encodes ASKη/BIN2 have shown that this protein is involved in the cross-talk between brassinosteroid and auxin signaling pathways [15]. Furthermore, a direct modulation of Auxin Response Factor 2 transcriptional activity by BIN2 has been revealed, uncovering a direct molecular link between auxin and BR signaling [21]. Recently, ASKtheta belonging to *Arabidopsis* GSKs clade III has also been involved in BR signaling [22], while evidence was provided for a possible implication of group I ASKgamma in this signaling pathway [20]. Consequently, so far, up to 5 out of 10 AtSKs belonging to 3 out of 4 clades are proposed to be involved in BR signaling.

Plant GSKs have been involved in a broad range of developmental processes such as embryonic, flower, stomata development as well as wound response. ASKdzeta, ASKeta/BIN2 and ASKtheta are expressed in developing embryos although their functions in embryonic development remain largely unknown [23,24]. Antisens *AtSKalpha* and *AtSKgamma* plants display a higher number of sepals and petals as well as alterations in the apical basal patterning of the gynoecium [25]. Brassinosteroid signaling is involved via BIN2 in stomata development [26]. Finally, the wound-induced GSK-3 (WIG) of alfafa participates in the wound response [27].

Considering the diversity of plant GSKs and the multifaceted functional capabilities already observed, it is essential to gain more insight on their role in plant development and to extend the studies to other plant families than *Brassicaceae*. In this report, we focused on the monocot *Poaceae* species due to their agronomical and ecological importance, phylogenetic relevance as well as their development in particular their embryonic development being in many aspects different from dicot *Arabidopsis* development.

In this article, we report the molecular characterization of two homolog wheat GSKs called *TaSK1* and *2* (*Triticum aestivum Shaggy like Kinase 1* and *2*) as well as their homoeologous gene copies. Chromosomal localization of the respective homoeologous gene copies and functional *in vitro* kinase activity for both homologs are provided. Furthermore, phylogenetic relationship of TaSKs to other relevant *Poaceae* GSKs and to selected dicots including the *Arabidopsis* ASKs is analyzed as a first step to provide a framework towards functionality studies.

## Results

### Molecular characterization of wheat *TaSKs*

A cDNA fragment encoding a protein with high identity to the mammalian Glycogen synthase kinase 3 (GSK-3) and to the *Drosophila* serine/threonine kinase SHAGGY (SGG) was isolated in the screen of an embryonic cDNA library constructed by means of a suppression subtractive hybridization (SSH) approach [28]. Using this fragment, two new cDNA sequences were obtained by SMART RACE cDNA amplification and named *Triticum aestivum Shaggy-like Kinase 1* and *2* (*TaSK1* and *TaSK2).* As a part of the 5′ of *TaSK1* could not be cloned by means of the latter technique, additional cloning was performed. Thus cloning followed by alignment of 23 cDNA and 56 genomic clones of *TaSK1* as well as 18 cDNA and 21 genomic clones of *TaSK2* provided evidence for the occurrence of three expressed gene copies of *TaSK1* and *TaSK2* named *TaSK1-A,B,C* and *TaSK2-A,B,C.* A manual approach and the algorithms/programs CLUSTALX, MAFFT, MUSCLE, Figtree and Quicktree were utilized to assemble, align and subgroup these cloned sequences [29-32]. Genomic and cDNA consensus sequences of *TaSK1-A*,*-B* and *-C*, and of *TaSK2-A*,*-B* and *-C* were extracted from the alignments.

Consensus genomic sequences of *TaSK1-A,B,C* had a size of respectively 4436, 4422 and 4195 bps. Intron 1 of *TaSK1-C* could not be cloned. The sizes of the consensus genomic sequences of *TaSK2-A,B,C* were 3825, 3999, and 3824 bps respectively. Their genomic structure is similar to the one reported for *Arabidopsis ASK*s [12] namely 12 exons interrupted by 11 introns.

Comparison of the three *TaSK1* consensus sequences at the genomic and cDNA level pointed out their high level of sequence conservation. Indeed, identity of *TaSK1-A,B,C* genomic sequences were ranging from 88.5 to 96% while the percentages of identity of their complete coding region (CDS) sequences were ranging from 96.9 to 98.4% (Table 1-A,B). Similarly, high identities were also observed for *TaSK2-A,B,C* genomic sequences (90.3 to 97.9%) and CDS sequences (98.9 to 99.7%) (Table 1-A,B).

**Table 1:** ***TaSKs*/TaSKs sequence identities at the genomic, CDS and protein level**

A						
Genomic	*TaSK1-A*	*TaSK1-B*	*TaSK1-C*	*TaSK2-A*	*TaSK2-B*	*TaSK2-C*
*TaSK1-A*		96.0	*88.8*	54.2	51.9	54.4
*TaSK1-B*			88.5	53.8	51.6	54.1
*TaSK-1C*				53.1	51.0	53.3
*TaSK2-A*					90.3	97.9
*TaSK2-B*						90.7
*TaSK2-C*						
B						
CDS	*TaSK1-A*	*TaSK1-B*	*TaSK1-C*	*TaSK2-A*	*TaSK2-B*	*TaSK2-C*
*TaSK1-A*		98.4	97.2	82.7	82.7	82.7
*TaSK1-B*			96.9	82.5	82.5	82.5
*TaSK1-C*				83.0	83.0	83.0
*TaSK2-A*					98.9	99.7
*TaSK2-B*						98.9
*TaSK2-C*						
C						
protein	TaSK1-A	TaSK1-B	TaSK1-C	TaSK2-A	TaSK2-B	TaSK2-C
TaSK1-A		99	99	88.3	88.6	88.6
TaSK1-B			98.8	88.3	88.6	88.6
TaSK1-C				88.6	88.8	88.8
TaSK2-A					99.3	99.3
TaSK2-B						99.5
TaSK2-C						

Indicated values represent the percentage of identity among the consensus sequences of *TaSKs* / TaSKs at the genomic level (A), CDS level (B) and predicted protein level (C). Identities were calculated using a Percent Identity Matrix created by Clustal 2.0.12. Sequence of *TaSK1-C* intron 1 is still unknown. Therefore genomic *TaSK1-C* identities were calculated by considering unknown intron 1 as being a gap.

TaSK1-A,C and TaSK1-B predicted proteins considering the longest open reading frame (ORF) contained respectively 400 and 401 amino acids. Their calculated molecular weight was respectively 44.9 and 45.0 kDa. The three *TaSK2* consensus cDNAs encoded predicted proteins of 402 amino acids (longest ORF) with a molecular weight of 45.2 kDa. Identity among TaSK1-A,B,C was ranging from 98.8 to 99% while identities among TaSK2-A,B,C were ranging from 99.3 to 99.5% (Table 1C). In comparison, TaSK1 and TaSK2 displayed 88.3 to 88.8% identity (Table 1C). Consequently, identities among the three TaSK1 and among the three TaSK2 were higher as the ones between TaSK1 and TaSK2.

### Copy number and chromosome localization of *TaSK* genes in the hexaploid wheat genome

Global alignment provided strong evidence for the presence of three expressed gene copies of *TaSK1* as well as three expressed copies of *TaSK2*. The complexity of the hexaploid wheat genome gives rise to the question whether these copies were homoeolog gene copies and/or paralog genes.

Nullisomic-tetrasomic lines of *Triticum aestivum* cultivar Chinese Spring have been successfully used to localize homoeologous genes on particular chromosomes of hexaploid wheat [33]. Such a line is missing a pair of chromosomes that is replaced by an extra pair of homoeologous chromosomes. In other words, these lines are nullisomic (N) for one of the homoeolog chromosomes, and tetrasomic (T) for one of the two other homoeolog chromosomes. Among the 42 tetra-nullisomic combinations possible, 19 combinations have been tested, the other ones being either redundant or lethal. In particular, nullisomic 2A and 4B lines were not available. Based on the known cloned sequences from wheat cultivar Sonora, specific primers for each gene copy were designed (Table 2). For each primer combination, one primer was designed to recognize a sequence region with a nucleotide insertion or deletion specific for a given gene copy, the second primer binding to an unspecific region of either *TaSK1* or *TaSK2*.

**Table 2:** **Specific primers used for the amplification of *TaSK *sequences in Nullisomic-Tetrasomic lines**

**Primers**	**Sequences (5′-3′)**	**Target sequences**
SF97	AGGCACATGATCAGTTCAATAAT	*TaSK1-A*
SR96	TATGTCTCCACCTCCTACATC	*TaSK1-A*
SF61	CCCAACGCAAGAGCAAAG	*TaSK1-B*
SR98	CCAGACGCGACATGAAATC	*TaSK1-B*
SF99	CTGCTAATGTATGTATCATCTGCT	*TaSK1-C*
SR101	AGGACATACTCGCAAGACTC	*TaSK1-C*
SF104	GATGTTACTTACCTATCATTTTTCTTGT	*TaSK2-A*
SR105	ACCTTGTGCCAAGGATGAG	*TaSK2-A*
SF102	CTCTTTAGCTATGACAACTCATTGA	*TaSK2-B*
SR102	TGAAAGACGATGCCAAACG	*TaSK2-B*
dCAPS-T2C-F	GAGCTGCAGCTTATGCGTTCG	*TaSK2-C*
dCAPS-T2C-R	AGATAAGTGGCATCCCCTGTTTGG	*TaSK2-C*

**Figure 1:**
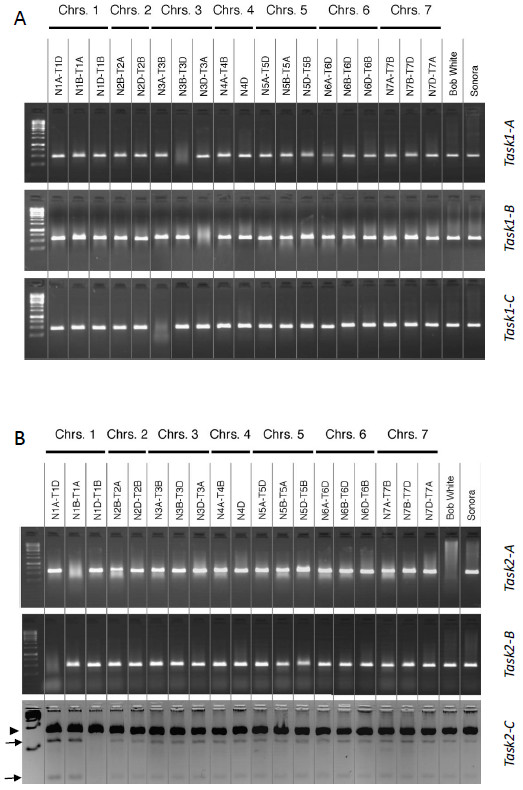
**Chromosomal localization of *TaSKs. *A**: chromosome localization of *TaSK1-A,-B,-C*. PCR on tetrasomic-nullisomic and nullisomic lines (cv Chinese Spring ) as well as control wheat genomic DNA (cv Bobwhite and Sonora) performed with primers specific for *TaSK1-A* (upper agarose gel), for *TaSK1-B* (middle agarose gel), and for *TaSK1-C* (lower agarose gel). **B**: chromosome localization of *TaSK2-A,-B,-C*. PCR on tetrasomic-nullisomic and nullisomic lines (cv Chinese Spring ) as well as control wheat genomic DNA (cv Bobwhite and Sonora) performed with primers specific for *TaSK2-A* (upper agarose gel), and for *TaSK2-B* (middle agarose gel). Lower agarose gel shows the result of the PCR performed using specific primers to amplify *TaSK2* sequences followed by a *Rsa1* endonuclease digestion, this digestion site being specific to *TaSK2-C*. Arrows show the fragments resulting from the *Rsa1* digestion of *TaSK2-C* amplicons. Arrowhead indicates the non-digested amplicons of *TaSK2-A* and *TaSK2-B*. N:nullisomic; T:tetrasomic; the letters A,B,D in the line name indicate the three genomes of hexaploid *Triticum aestivum*.

A PCR-product was obtained with specific primers for *TaSK1-A* in all lines tested except in the line N3B-T3D providing strong evidence for the localization of *TaSK1-A* on chromosome 3B (Figure 1A). Amplification was obtained with primers specific for *TaSK1B* for all lines except N3D-T3A strongly suggesting a localization of *TaSK1-B* on chromosome 3D (Figure 1A). Similar approaches led to the conclusion that *TaSK1-C* most probably is located on chromosome 3A as the only line without amplification is N3A-T3B (Figure 1A).

Similarly, *TaSK2-A* probably localizes on chromosome1B while *TaSK2-B* is located on chromosome 1A (Figure 1B). Unfortunately such a Polymerase Chain Reaction (PCR) approach based on sequence specific primers was not suitable for *TaSK2-C* as this sequence did not have an appropriate specific nucleotide insertion or deletion that distinguished it from the other ones. However exon 4 of *TaSK2-C* carried a conserved nucleotide exchange that created an *Rsa1* restriction site absent in the other *TaSK2* and in *TaSK1* sequences. Specific primers for *TaSK2* sequences were designed upstream and downstream of this restriction site (Table 2). Amplification followed by *Rsa1* digestion gave rise to a digestion product in all lines tested (arrows) except in line N1D-T1B suggesting that the digestion site was absent in this line (Figure 1B). We therefore conclude that *TaSK2-C* is located on chromosome 1D.

In summary, *TaSK1-A,B,C* were located on the homoeologous chromosomes 3B, 3D, 3A while *TaSK2-A,B,C* were located on the homoeologous chromosomes 1B, 1A, 1D. Most probably each gene copy was present only on one chromosome. These data strengthen the results of the sequence alignment analysis and provided strong evidence that the three *TaSK1* copies on one hand and the three *TaSK2* copies on the other hand were homoeolog gene copies.

### *Triticum aestivum* TaSKs display a GSK3/SGG signature

The catalytic domains of TaSK1-A,B,C and TaSK2-A,B,C shared high sequence identity with the catalytic domains of *Arabidopsis thaliana* BIN2 (91-90%), *Drosophila melanogaster* SHAGGY (67-68%) and *Homo sapiens* GSK-3β (70-71%). As comparison, human GSK-3β and *Drosophila* SHAGGY showed 85% identity in their catalytic domain. The mentioned percentages were pairwise alignment scores obtained by means of ClustalW2.

**Figure 2:**
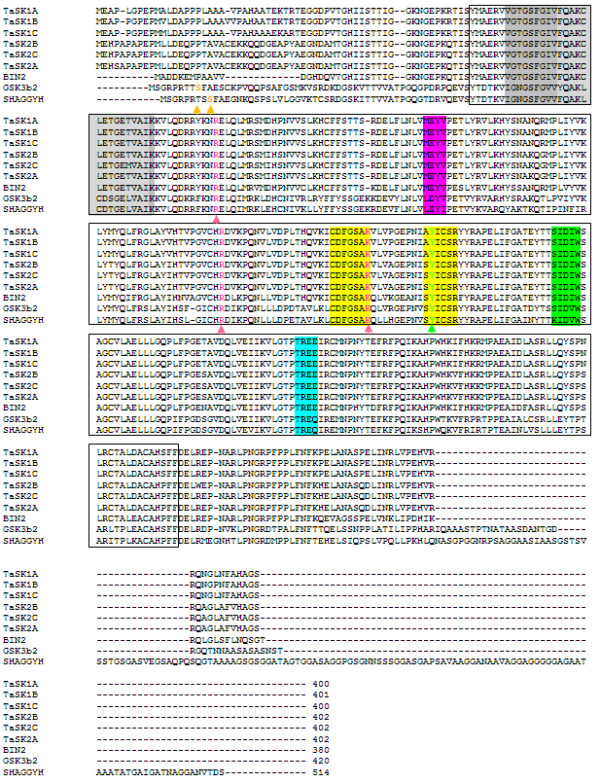
**Alignment of predicted TaSKs amino acid sequences with selected animal and plant GSKs.** TaSK1-A,B,C and TaSK2-A,B,C have been aligned with the *Homo sapiens* GSK3-β ([GenBank: NP_001139628], major and shorter splice variant), the *Drosophila melanogaster* SHAGGY isoform H [GenBank: AAS65253] and the *Arabidopsis thaliana* BIN2 [TAIR: AT4G18710]. Full length protein alignment was performed using ClustalX 2.0.12 software. The catalytic domains of the protein kinases as defined by Hanks and Quinn (1991) [34] are framed in black. The highly conserved motifs CDFGSAK and SYICSR, present only within members of the GSK-3 subfamily of serine/threonine protein kinases are boxed in yellow. The grey box highlights the ATP-binding region. The motif SIDIW present only in members of plant group II GSKs and the animal/human motifs in equivalent position are boxed in green. The blue background highlights the TREE motif of plant GSKs and the motifs present in equivalent position in animal/human GSKs. The MEYV motif that contains key residues for binding of plant GSK inhibitor Bikinin and the corresponding animal motifs are boxed in pink. Arg 96, Arg 180 and Lys205 residues of human GSK-3β defining the pocket for primed substrate binding and being present in equivalent positions in plant GSKs are marked in pink (pink arrowhead). Tyr 216 in human GSK-3β present in equivalent positions in plants and in *Drosophila* GSKs is marked in green (green arrowhead). Ser 9 present in animal GSKs but absent in TaSKs is highlighted in orange (orange arrowhead).

All TaSKs contained the highly conserved motifs CDFGSAK and SYICSR (AYICSR in the case of TaSK2s) that distinguish the GSK-3 subfamily among serine/threonine protein kinases (Figure 2) [23,34].

Within the latter motif, all TaSKs had a tyrosine (Tyr) residue in equivalent position to the Tyr 216 of GSK-3β (Figure 2). Phosphorylation of this residue is implicated in the modulation of kinase activity in human and in *Arabidopsis*[20,35-37].

Residues in equivalent position to Arg 96, Arg 180, Lys 205 of GSK-3β were present in TaSKs (Figure 2). In the case of GSK-3β, these residues define a pocket for binding of primed substrates [35,36,38]. Pre-phosphorylated (primed) substrate by another kinase binds to this pocket and is thereby correctly positioned for a phosphorylation by GSK-3β [35,36,38]. However, although *Arabidopsis* BIN2 contains this pocket, its phosphorylation activity is not based on priming phosphorylation but rather requires a direct interaction with BRZ1 [39].

Inhibition of GSK-3β in the insulin signaling pathway relies on N terminal residue serine 9 (Ser9) phosphorylation [38]. Ser9 was absent in TaSKs as it is the case for BIN2 (Figure 2) [7].

TaSKs were classified in group II due to the presence of the SIDIW box characteristic for plant group II GSKs (Figure 2) [23]. Like *Arabidopsis* BIN2, TaSKs contained the TREE motif (Figure 2). Almost all *Bin2.1* gain of function mutations localize to this motif [13,15,19]. *Bin2.1* protein was shown to be more stable than its wild type form [19]. TaSKs contained also the motif MEYV that contains key residues for docking of *Arabidopsis* GSK inhibitor Bikinin (Figure 2) [40]. This inhibitor poorly inhibits human GSK-3β whose motif in equivalent position is LDYV [40].

Protein sequence analysis provides strong evidence for a classification of TaSKs in the GSKs subfamily of protein kinases.

### TaSK1 and TaSK2 are functional kinases

**Figure 3:**
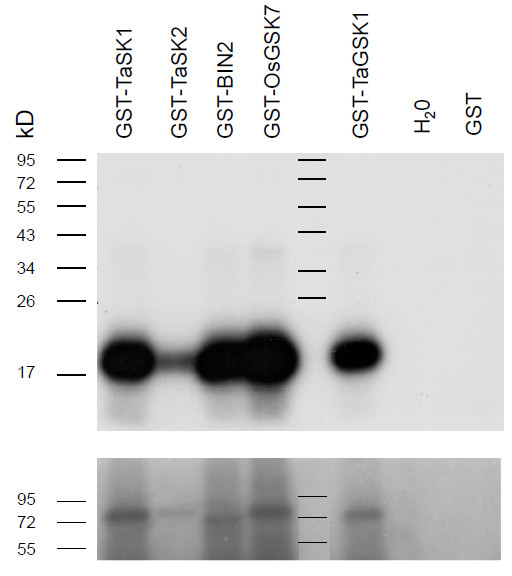
***In vitro *kinase assays.** Activity of the purified kinases was determined by *in vitro* kinase assays using γP^32^ATP. An equal volume of GST-TaSK1 (71,585 kDa), GST-TaSK2 (71,917 kDa) , GST-BIN2 (69,877 kDa), GST-OsGSK7 (72,174 kDa), GST-TaGSK1 (70.154 kDa), and GST (27,898 kDa) purified fusion proteins was used to test their phosphorylation activity on a myelin basic protein fragment (18,454 kDa). After kinase reaction, samples were loaded on a 12% SDS PAGE gel. After migration, the gel was directly exposed to an X-ray film for either 80 minutes (upper panel) or 24 hours (lower panel). OsGSK7 and BIN2 locus names are respectively RGAP: LOC_Os05g11730 and TAIR: AT4G18710, while the accession of TaGSK1 is GenBank: AF525086.

An *in vitro* kinase activity assay was performed to evaluate whether TaSK1 and TaSK2 had a kinase activity. Full length TaSK1 and TaSK2 (longest ORF), *Arabidopsis* BIN2, OsGSK7 (TaSKs homolog in *Oryza sativa*) and wheat TaGSK1 were overexpressed in *E. coli* as GST fusion proteins and affinity purified in native conditions. Transphosphorylation activities towards a bovine myelin basic protein fragment (MBP) that contains several consensus phosphorylation sites for kinase proteins was assayed using radiolabeled ATP (Figure 3). TaSK1, TaSK2, OsGSK7, BIN2 and TaGSK1 expressed as fusion proteins were phosphorylating the MBP fragment while GST alone was not able to phosphorylate MBP (Figure 3, top panel). Furthermore, a long exposure of SDS-PAGE gel to the X-ray film showed a clear autophosphorylation activity for TaSK1, BIN2, OsGSK7 and TaGSK1 (Figure 3, lower panel) while a weaker signal was observed for GST-TaSK2.

These data indicate that cloned TaSK1 and TaSK2 were functionally active kinases.

### TaSK1 and TaSK2 belong to clade II of plant GSKs

The genome of the core eudicotyledonous *Brassicaceae Arabidopsis thaliana* contains 10 different GSKs. These ASKs have been grouped based on their sequences into either three [12] or four [41] major clades. Little is known about Liliopsida, resp. *Poaceae* GSKs and their phylogenetic relationship in comparison to *Arabidopsis*. Therefore, the phylogenetic relationship of GSKs of selected *Poaceae* to *Arabidospsis* ASKs and to GSKs belonging to other selected eudicotyledons was investigated and analysed.

The *Poaceae* family includes 12 subfamilies among them the *Pooideae, Ehrhartoideae* and the *Panicoideae* that provide the bulk of human nutrition. Besides *Triticum aestivum* (wheat), the *Pooideae* contain species like *Hordeum vulgare* (barley) and *Brachypodium distachyon* the latter being proposed recently as new grass model system [42]. *Oryza sativa* (rice) and *Zea mays* (maize) were selected as representatives of the *Ehrhartoideae* and *Panicoideae,* respectively.

*Arabidopsis thaliana* is a paleopolyploid that has been subjected to two additional whole genome duplications (WGDs) called α and β events after the so-called γ event, the latter being a triplication event resulting in the hexaploid common ancestor of many or most angiosperms [43-45]. Therefore, the GSK sequences of two core eudicotyledons, the Brassicale *Carica papaya* and the basal Rosid *Vitis vinifera,* both not exposed to the more recent α and β WGD events specific to *Brassicaceae* or to any other events after the γ duplication*,* were included in this study [46-48]. In addition, GSK sequences of *Aquilegia coerulae* a member of the basal-most or stem eudicotyledons (Ranunculales) and the moss *Physcomitrella patens* as representative of non-seed plants were added to this phylogenetic analysis. The genomes of all these plants are fully sequenced.

Except for published rice GSK and *Arabidopsis* ASK gene sequences [7,41], the sequences of the other GSKs were identified in different databases by means of annotation mining and BLAST (Basic Local Alignment Search Tool) searches (Additional file 1).

Besides TaSK1-A,B,C and TaSK2-A,B,C only 4 other wheat GSK sequences have been identified in the databases. Considering the complexity of the wheat genome due to its size (16,000 Mb) and polyploidy, this number appears low. Probably more sequences will be identified once full genome sequencing data will be available in open access databases. Twenty-eight different maize GSKs were found in the databases (Additional file 1). However, among them 10 subgroups were distinguished in which identities between the predicted proteins were ranging from 97 to 99%. Maize is diploid and the tissues used to generate the cDNAs were derived from different hybrids [49,50]. Therefore, we hypothesized that the predicted proteins within each group may be the same and that the difference observed may be due to strain polymorphism or might be caused by sequencing artifacts inherent to high throughput sequencing approaches, respectively difference in gene structure prediction. As a consequence, one accession within each group was chosen as representative for this group (Additional file 1).

**Figure 4:**
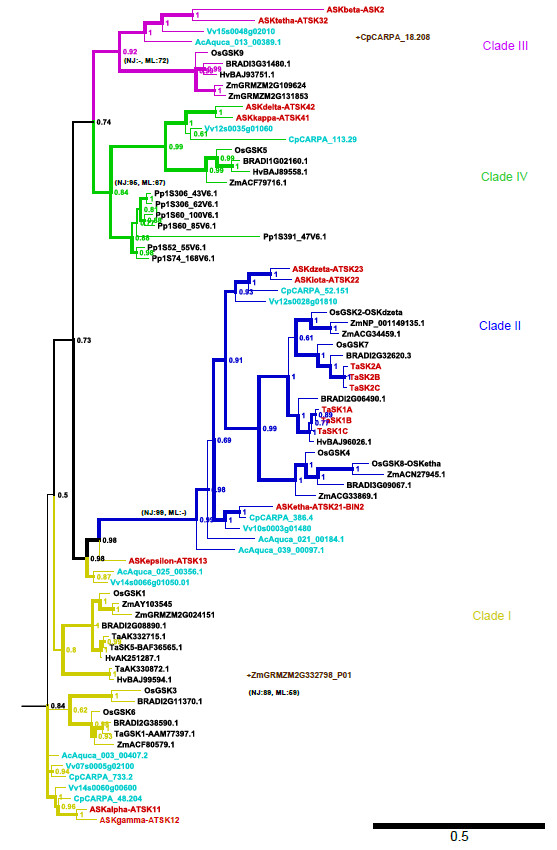
**Phylogenetic tree of land plant shaggy-type kinases.** Bayesian inference topology based on a curated full length amino acid sequence alignment. The tree is outgroup rooted, numbers at the nodes are posterior probabilities (in addition, bootstrap and quartet puzzling support values are shown for the four colored subfamily clades); line width also reflects support by Bayesian inference. *A. thaliana* SHAGGY kinases and the six *T. aestivum* kinases reported here are shown in red, sequences from the three eudicotyledons that have not been subjected to genome duplications beyond the γ event (*C. papaya*, *V. vinifera* and *A. coerulea*) are shown in cyan. For two truncated gene models excluded from the analysis the approximate position (to which clade they belong) is shown in brown.

As already mentioned, previous phylogenetic analyses led to the identification of different plant GSK clades named I to IV [12,41]. This clade nomenclature was kept here based on the *Arabidopsis* and rice GSK clade members. With regard to these previously defined subfamilies, the Neighbour-Joining (NJ) tree supported clade II (in blue), clade IV (green) and clade I (yellow) as monophyletic with bootstrap support 89–99 (Additional file 2), while the Maximum Likelihood (ML) tree supported clade III (purple), clade IV (green) and clade I (yellow) with 59–72 quartet support (Additional file 3) and the Bayesian Inference (BI) tree supported the clade III (purple), clade II (blue) and clade IV (green) with posterior probabilities of 0.84-0.99 (Figure 4). Thus, clade IV (green) is supported by all three methods, while the blue (II), yellow (I) and purple (III) clades were supported as monophyletic by two out of three methods each.

In all three trees, TaSK1-A,B,C and TaSK2-A,B,C sequences clustered reliably in respectively two closely related subclades together with related sequences from other grasses (Figure 4, Additional file 2 and 3). In the BI and NJ trees, these two subclades containing TaSK1-A,B,C and TaSK2-A,B,C were embedded into a monophyletic clade II (blue) (Figure 4, Additional file 2). Interestingly clade II includes ASKeta/BIN2, and its close relatives ASKdzeta and ASKiota (Figure 4)*,* all three being involved in brassinosteroid signaling [51]. None of the wheat GSKs identified so far in the databases was classified in clade III that includes ASKtheta shown to be also involved in brassinosteroid signaling [22]. Nevertheless all the other *Poaceae* selected had at least one GSK representative in this clade (Figure 4).

The presence of the *Physcomitrella patens* paralogs exclusively at the base of the green clade, supported by all three analyses, suggests that the clade IV (green) was the ancestral one (present already in the earliest land plants), from which all other family members have subsequently evolved (Figure 4, Additional file 2 and 3). Interestingly, lineage-specific gain and retention of paralogs have occurred in *Physcomitrella patens*, bringing the extant number of paralogs to seven, comparable to the numbers in seed plants (see below). Three paralog pairs are probably derived from the WGD known to have occurred in this lineage [52].

The general theme as revealed by the phylogeny is that each of the eudicotyledon species is represented with one (green, purple), two (blue), or two/three (yellow) sequences per subfamily (Figure 4). In the green (ancestral) clade no *A. coerulea* sequence is present. Most likely, this sequence has been subject to a secondary loss or there is no adequate gene model present for this sequence. The purple clade lacks a *C. papaya* sequence, however, this sequence has been removed due to a truncated gene model (cf. Methods). The yellow clade harbours three *V. vinifera* genes and two each of the other three species, the tree topology suggesting secondary loss of the latter two (Figure 4). In summary, these results point out the presence of 6 (possibly 7) GSKs in the ancestral eudicotyledonous genome after the gamma event, resp. after the separation from the Liliopsida.

The phylogeny of the Liliopsida, considering fully sequenced genomes (all except *H. vulgare* and *T. aestivum*), indicate that each of these species has in general one (purple, green), three (yellow) or three-/four (blue) GSK sequences per clade. Clade I (yellow) does not include a third *Zea mays* GSK. This sequence has been removed because it was incomplete (cf. Methods). A fourth rice GSK was identified in clade II. Taken together these data indicate that 8 GSKs were present in the genome of the Liliopsida-ancestor at the time it diverged from the eudicotyledons.

## Discussion

Little is known about wheat non-receptor serine/threonine kinases. To the best of our knowledge, *TaGSK1* involved in salt tolerance was the only member of this multigene family investigated so far in wheat [53]. We identified two expressed gene sequences called *TaSK1* and *TaSK2* in the wheat genome. Protein sequence analysis of TaSKs clearly indicated a GSK signature. In particular, they both have a tyrosine residue in equivalent position to Tyr 216 of GSK-3β whose phosphorylation status modulates kinase activity. Dephosphorylation of the equivalent BIN2 tyrosine residue (Tyr200) by BSU phosphatase, the latter being a positive regulator of BR signaling, inhibits the kinase activity of BIN2 [20]. In humans, the phosphorylation of this tyrosine residue located in the activation loop is proposed to facilitate substrate binding by making easier binding site accessibility [35,36]. Although phosphorylation of Tyr 216 is not strictly required for kinase activity, it is proposed to increase notably the catalytic activity of GSK-3β [35,37]. TaSKs contain also residues in equivalent positions to Arg 96, Arg 180, Lys 205 of GSK-3β although the relevance of these residues for the activity of TaSKs remains to be clarified. In the case of GSK-3β, these residues create a pocket for binding of primed substrates. GSK-3β has a preference for primed substrates that are previously phosphorylated by another kinase at the priming phosphorylation site located four amino acids C terminal to the site of GSK phosphorylation [35,36,38]. Binding of primed substrates to this pocket is proposed to position them correctly in the catalytic groove for subsequent phosphorylation by GSK-3 and to stabilize the active conformation of the enzyme [35,36,38]. In animals, a tight kinase-substrate docking interaction can also be achieved by a different mechanism involving a scaffold protein binding simultaneously GSK3 and its substrate [6]. Requirement for this pocket appears to be different in human and in plants. Although BIN2 contains the pocket for binding of primed substrates, the kinase has been shown to interact directly with BZR1 via a mechanism different from the two common docking mechanisms described in mammalians [39]. Ser9 residue whose phosphorylation leads to the inhibition of GSK-3β in the insulin pathways [38] is absent in TaSKs as it is the case for BIN2 [7]. Phosphorylation of Ser 9 residue produces a primed pseudo-substrate that binds intramolecularly to the pocket for primed substrate binding, thereby hindering in a competitive manner phosphorylation of true substrates by GSK-3β [35,36,38]. Inhibition of TaSKs consequently must rely on another mechanism.

*In vitro* kinase activity assays showed that TaSKs were functionally active kinases. In addition, they were also capable of autophosphosphorylation. Autophosphorylation has also been observed for BIN2 and ASKtheta [22,54]. Tyr 200 of BIN2 has been identified *in vitro* by mass spectrometry as a major autophosphorylation site [20]. Mutation of Tyr 200 to Phe greatly reduces the phosphorylation of the substrate of BIN2 [20]. Similar effects were also observed for human GSK-3 [35,37]. However, the functional relevance of the autophosphorylation of TaSKs remains to be elucidated.

*TaSK1* and *TaSK2* predicted proteins shared identities ranging from 88.3 to 88.8%. For each gene, three gene copies located on homoeologous chromosomes were identified. Indeed, chromosome localization using tetrasomic-nullisomic lines unraveled that *TaSK1-A,B,C* were located on chromosome 3B, D and A while *TaSK2-A,B,C* were identified on chromosome 1B, A and D. Identities among predicted proteins encoded by *TaSK1-A,B,C* were ranging from 98.8 to 99% while proteins encoded by *TaSK2-A,B,C* displayed 99.3 to 99.5% identity*.*

Evolutionary history of hexaploid wheat includes two polyploidizations events [55]. In a first step about 0.5-0.36 million years ago, hybridization occurred between two diploid species *Triticum urartu* (genome A^u^A^u^) and most probably *Aegilops speltoides (genome SS, close to BB).* Hexaploid *Triticum aestivum* originated by the hybridization of cultivated tetraploid wheat *Triticum turgidum* (genome *BBAA*) with diploid *Aegilops tauschii (genome DD)* about 10.000 years ago.

Interestingly *TaSK1-A and –B,* the two closest gene copies among the *TaSK1,* as well as *TaSK2-A and –C,* the two closest copies among the *TaSK2,* were located on genome B and D to which the two *Aegilops* species contributed.

Thus, *TaSKs* are a perfect example for the complexity of biological systems. They belong to a multigene family known to encode multitasking proteins and they are represented in wheat by three homoeologous gene copies each. A very interesting although challenging question to be addressed in this context is the relevance of *TaSK* homologs and homoeologs in terms of sub-, neo- or even non-functionalization.

This question is of special interest in the light of homoeolog gene expression biases observed in the allopolyploid *Gossypium*[56,57]. The study of Flagel *et al.,* (2008) [57] showed that for a large fraction of cotton genes contributing to the petal transcriptome, this bias resulted from long-term evolutionary processes including neofunctionalization and subfunctionalization of duplicated genes. For a smaller fraction of genes, biased expression patterns were proposed to have occurred immediately with polyploidization as a consequence of the genomic merger. Adam *et al.* (2003) [56] observed that a significant number of analyzed cotton genes showed a developmentally regulated silencing or biased expression. A reciprocal silencing of homoeologs in different organs was reported such that both genes remain functional in different parts of the plant, suggesting subfunctionalization.

Although plant GSKs may be produced by differential transcription, remarkable is that they are encoded by a multigene family. In contrast, human genome contains three GSK3 isoforms encoded by only two genes [58,59] while different isoforms of *Drosophila* SHAGGY originate by alternative splicing from a single gene [60]. Larger numbers of genes in plants compared to animals are also observed for other proteins such as the MADS-box transcription factors [61]. Plants have apparently more predisposition than animals to gene duplication followed by functional diversification [61,62].

Phylogenetic relations pointed out that both TaSKs were members of GSK group II to which belong ASKiota, ASKdzeta and BIN2. These ASKs are all three involved in brassinosteroid signaling [13,14]. Studies of multigene encoded families such as MADS box proteins propose a strong correlation between primary structure and regulatory functions [61]. This raises the question whether the belonging of monocot TaSKs to group II correlates with a function in BR signaling as shown for *Arabidopsis* group II ASK.

The number of ASKs involved in brassinosteroid signaling indicates a high redundancy. However, the binding specificity of ASKtheta and BIN2 to transcription factors of the BRZ1/BES1/BEH2 family is different for each factor [22]. In addition, the expression patterns of BIN2, ASKdzeta, ASKiota and ASKtheta are distinct, although to some extent overlapping [23,24]. Despite redundancy, these observations point towards a certain functional specialization in brassinosteroid signaling.

TaSK1 and TASK2 displayed a high identity at the protein level. Amino acid sequence analysis indicated that all motifs and residues identified in plants or in animals which are relevant for the function, classification, inhibition, or stability were identical in both proteins. The only exception is the amino acid next to the functional Tyr in equivalent position to Tyr 216 of GSK-3β and to Tyr 200 of BIN2. This residue was in TaSK1 an alanine instead of a serine. Interestingly, one *Physcomitrella* GSKs out of seven displayed also this change of amino acid. First hints about a possible functional specialization of TaSK1 towards TaSK2 may be given by developmental, organ as well as subcellular expression pattern.

Angiosperms underwent whole genome duplications (WGD) early in their evolution, so called paleoploidizations [63]. The γ WGD is a triplication event that resulted in the hexaploid common ancestor of many or most angiosperms. The placement of the γ event is still unclear. Different split points have been proposed, namely (1) before the separation of eudicotyledons and Liliopsida, (2) in a common ancestor of all eudicotyledons, (3) before the separation of rosids and asterids, and finally (4) as a rosid wide duplication [64]. Recently, strong evidence has been provided for an occurrence close to the core eudicotyledon diversification (after the split of Liliopsida and eudicotyledons, and before the separation asterid-rosid) [64]. It was furthermore hypothesized that two additional WGD occured, one in the common ancestor of seed plants and the other one in the common ancestor of all angiosperms, both predating the gamma event [45]. In addition, more recent α and β WGD events occurred within the *Brassicaceae*[44,45]*.* A polyploidy event called σ was proposed to have taken place in the Liliopsida lineage after the divergence from the eudicotyledons and the more recent event ρ occurred in the cereal lineage preceding the radiation of their major cereal lineages [65].

Our phylogenetic analyses, based on the presence of lineage-specific in-paralogs, showed that many genes in *A. thaliana* and the grasses were derived from WGD that were more recent than the γ event, from segmental duplications, or from recent polyploidization. If one disregards these paralogs/homoeologs, clade III and IV contain a single gene each. Clade I and II show evidence of two or three ancestral paralogs for respectively the dicotyledon or Liliopsida species. However, the ancestral situation in clade I might have been three paralogs for dicotyledons as outlined above, which might be convoluted by secondary losses. The fact that the Liliopsida (here: grasses), the stem eudicotyledons and core eudicotyledons all showed evidence for the same ancestral set of six to seven genes allows for two possible explanations: i) the paralogs are derived from the γ event, in which case it would have occurred prior to the Liliopsida/eudicotyledon split or ii) the paralogs were retained after one or both of the more ancient WGD mentioned above. To determine which scenario is more likely is beyond the scope of this study.

TaSK1-A,B,C and TaSK2-A,B,C clustered in respectively two closely related subclades. The two distinct subfamilies were most probably derived from the last whole genome duplication (ρ event) common to the grasses [45,65], while the three sequences in each cluster most probably represent homoeologs from recent polyploidization as already inferred consequently to their localization on homoeolog chromosomes.

Retention of genes after large scale duplication has been proposed to be biased dependent to the function of genes [66,67]. Indeed, genes important in development, transcriptional regulation and signal transduction - thus major players in biological complexity and morphological diversity - are proposed to have a higher probability to be retained after land plant WGD events [63,68].

## Conclusion

Two GSK homologs in the hexaploid wheat genome*, TaSK1* and *TaSK2* with 88% identity on the protein level, were identified and characterized. Their homoeologous gene copies were localized respectively on the homoeologous chromosomes 3B, 3D, 3A and on the homoeologous chromosomes 1B, 1A, 1D. TaSKs displayed all motifs and residues identified in plants or animals described as relevant for GSK function, classification, inhibition, or protein stability. Kinase and autokinase activity of the respective GST fusion proteins was tested and confirmed by *in vitro* kinase assays. Phylogenetic analysis revealed that both belong to GSK clade II, the *Arabidopsis* members of which are all involved in brassinosteroid signaling. Based on a single ancestral shaggy-like kinase in the last common ancestor of all land plants, paralogs were acquired and retained by ancestral WGD events, bringing the base number in angiosperms to six - eight. More recent WGD or segmental duplication events have increased the number up to ten in some lineages.

These findings lay the foundations to explore Liliopsida resp *Poaceae* GSKs functions in plant development. *TaSK* sequences included both paralogous and homoeologous gene copies allowing to address the relevance of these genes copies in term of sub, neo- or even non-functionalization. Knowledge gained on the molecular and phylogenetic level about TaSKs but also other selected *Poaceae* GSKs provides a framework to evaluate whether a function in BR signaling is evolutionary conserved among clade II angiosperm GSKs. Phylogenetic analysis shed light on acquisition and retention of GSK paralogs in angiosperms in the context of whole genome duplication events and provided information on the ancestral gene set of Liliopsida/eudicotyledon GSKs.

## Methods

### Cloning and sequencing of *TaSK* cDNA and genomic clones

A cDNA fragment of *TaSK1* was originally isolated by screening of an embryonic cDNA library constructed by means of suppression subtractive hybridization method (SSH). For the SSH, total RNA was isolated from embryo material of *Triticum aestivum* cv Sonora using TRIzol® Reagent (Invitrogen). Dynabeads Oligo (dT)_25_ from Dynal were used to purify mRNA. SSH was performed using the PCR-select cDNA substraction kit (Clontech) according to the suppliers instructions. 5′ and 3′ ends of the gene fragment were generated by means of BD SMART™ RACE cDNA Amplification Kit (Clontech) as recommended by the manufacturer.

Several cloning approaches were used to obtain genomic and cDNA sequences of *TaSK1*-*A,B,C* and *TaSK2-A,B,C*.

Total RNA and genomic DNA were extracted from *Triticum aestivum* cv Sonora tissues using respectively the RNeasy® Mini Kit and the DNeasy® Plant Mini Kit (QIAGEN) as recommended by the manufacturer.

Reverse transcription was performed using either the SuperScript™ II Reverse Transcriptase (Invitrogen), the RevertAid™ H Minus First Strand cDNA Synthesis Kit (Fermentas) or the *Bca*BEST™ RNA PCR Kit (Takara) as recommended by the manufacturers. Standard PCR procedure or PCR using *Bca*BEST™ RNA PCR Kit (Takara) were used to amplify the cDNAs.

Genomic DNA was amplified either by means of conventional PCR that may include the use of *Bca*BEST™ RNA PCR Kit (replacing cDNA by genomic DNA), inverse PCR [69] or thermal asymmetric interlaced PCR [70,71].

PCR products were cloned in the pCR®II-TOPO®, pCR®2.1-TOPO® and pCR®-Blunt® vectors (Invitrogen). Sequencing was outsourced to GATC Biotech AG, Agowa GmbH, Eurofins MWG GmbH, and Sequence Laboratories Göttingen GmbH.

Sequenced *TaSK1* / *TaSK2* cDNA and genomic clones were assembled, aligned, subgrouped manually and by means of CLUSTALX2.0.12, Multiple Alignment using Fast Fourier Transform (MAFFT) v6.717b, and MUSCLEv3.8 algorithms. The phylogenetic tree programs Figtree v1.3.1 and Quicktree-SD were used in addition to subgroup the cloned genomic and cDNA sequences.

Accession numbers for genomic and cDNA sequences deposited at the GenBank database are as follows: TaSK1A [GenBank: JX307288, GenBank:JX294419], TaSK1B [GenBank:JX307289, GenBank:JX294420], TaSK1C [GenBank:JX307290, GenBank:JX307292], TaSK2A [GenBank:JX307291, GenBank:JX307293], TaSK2B [GenBank:JX312689, GenBank:JX312688], TaSK2C [GenBank: JX312691, GenBank:JX312690].

### Chromosome localization of *TaSKs*

Nullisomic-tetrasomic lines (cv Chinese Spring) originally established by Sears *et al*. (1966) [33] were provided by the National small grains Germplasm Research facility of the United States Department of Agriculture (USDA).

Genomic DNA was extracted using the standard CTAB-DNA isolation [72].

Specific primers for amplification of *TaSK1-A, TaSK1-B and TaSK1-C* sequences were respectively SF97/SR96, SF61/SR98 and SF99/SR101 (Table 2). Specific primers for amplification of *TaSK2-A*, *TaSK2-B* and *TaSK2-C* were respectively SF104/SR105, SF102/SR102, dCAPS-T2C-F/dCAPS-T2C-R (Table 2). *TaSK2-C* amplicons were subsequently digested with *RsaI* endonuclease.

DNAs extracted from lines of cv Sonora and Bobwhite were used as control as the primers were designed based on cv. Sonora *TaSK* sequences.

### *In vitro* kinase activity assays

The N-Terminus of TaSK1, TaSK2, BIN2, OsGSK7 and TaGSK1 full length proteins were cloned in frame with a Gluthatione-S-Transferase (GST) tag.

GST-TaSK1, GST-TaSK2, GST-BIN2, GST-OsGSK7, and GST-TaGSK1 fusion proteins were overexpressed in *E. Coli.* and affinity purified in native conditions on Gluthatione Sepharose 4B resin. *In vitro* kinase reactions were performed by adding to 10 μl of the purified GST-fusion protein, 20 μl of the kinase activity buffer (20 mM HEPES, pH 7.4, 15 mM MgCl_2,_ 5 mM EGTA, 1 mM DTT) containing ATP γ^32^P and the bovine myelin basic protein (MBP, fragment) as described by Jonak *et al*., (2000) [27]. The reaction was incubated at room temperature for 45 minutes and subsequently stopped by adding 10 μl of SDS-Page loading buffer. After denaturation at 95°C for 1 minute, protein phosphorylation was analysed by autoradiography after migration on a 12% SDS/PAGE gel.

### Phylogenetic analysis

After the initial selection of GSK homologs by annotation mining and BLAST searches, homologs were detected using BLAST (cutoff 30% alignment identity and 80 amino acids alignment length) in selected genomes. The GSK sequences of selected lineages were obtained from EnsemblPlants, The Arabidopsis Information Resources (TAIR), Plant Genome DataBase (PlantGDB), Phytozome, GenBank, Hawaii Papaya Genome Project ASGPB, and the Rice genome annotation project (RGAP) databases. The current dataset contains only genes confirmed from completely sequenced genomes, except for *Hordeum vulgare* and *Triticum aestivum.* Identified sequences were analyzed for the presence at the protein or predicted protein level of the relevant GSK motifs and residues. Only *Poaceae* sequences with evidence at transcript level were included in this study. Curation of initial phylogenetic trees led to discarding duplicated sequences. Sequences not belonging to annotated shaggy kinases were separated by a long branch in the initial trees; all but two of the sequences were discarded, the remaining serving as outgroup. Protein accessions or locus name are listed in Additional file 1.

The multiple alignment of the full length amino acid sequences was generated using M.A.F.F.T. v6.717b [32] in the ‘auto’ mode and was subsequently manually curated (removing regions of poor alignment quality) using Jalview v2.7 [73], resulting in 501 columns that were used for phylogenetic inference. Two *Zea mays* [EnsemblPlants:GRMZM2G075992_P01, GRMZM2G332798_P01] and one *Carica papaya* [ASGPB: CARPA_18.208] sequences were not included in the phylogenetic analyses because the gene models appeared incomplete, covering less than 50% of the alignment, and the sequences thus were placed on very long branches in the initial phylogenies. Final topologies were inferred by Neighbour-Joining (NJ) using QuicktreeSD [30,74] with 1,000 bootstrap samples, by Maximum Likelihood (ML) using TreePuzzle v5.2 [75] with quartet puzzling and eight gamma distributed rates and by Bayesian Inference (BI) using MrBayes v3.1.2 with eight gamma distributed rates and two hot / two cold chains for two million generations. ProtTest v1.3 [76] was used to determine the model best suited to the dataset and turned out to be JTT with gamma distributed rates and invariant sites, which was hence applied for ML and BI inference. The trees were outgroup-rooted on the branch leading to two related, non-shaggy type *A. thaliana* kinases [TAIR: AT1G73690.1, AT1G67580.1].

## Authors’ contribution

TB carried out and contributed substantially to the experimental design of the molecular characterization*,* the copy number determination and chromosomal localization of *TaSKs*, as well as the *in vitro* kinase activity assay. SC performed the SSH and initiated the molecular characterization of *TaSK1* and *2.* GN was involved in results discussion. SAR made a substantial contribution to the phylogenetic analysis concerning experimental design and writing of the manuscript, and performed the Bayesian, Maximum Likelihood, and the Neighbour-Joining analysis. CFI conceived and coordinated the project, contributed significantly to the phylogenetic analysis, and wrote the manuscript. All authors read and approved the final manuscript.

## Supplementary Material

Additional file 1 List of plant GSKs included in the phylogenic analysis. Click here for file

Additional file 2 Neighbour-Joining tree. Click here for file

Additional file 3 Maximum Likelihood (ML) tree. Click here for file

## References

[B1] Oreña SJ Torchia AJ Garofalo RS Inhibition of glycogen-synthase kinase 3 stimulates glycogen synthase and glucose transport by distinct mechanisms in 3T3-L1 adipocytes J Biol Chem 2000 275 15765 15772 10.1074/jbc.M910002199 10748179

[B2] Cohen P Frame S GSK-3 takes centre stage more than 20 years after its discovery Biochem J 2001 359 1 16 10.1042/0264-6021:3590001 11563964 PMC1222116

[B3] Zumbrunn J Kinoshita K Hymann AA Näthke IS Binding of the adenomatous polyposis coli protein to microtubules increases microtubule stability and is regulated by GSK-3 beta phosphorylation Curr Biol 2001 11 44 49 10.1016/S0960-9822(01)00002-1 11166179

[B4] Jope RS Johnson G The glamour and gloom of glycogen synthase kinase-3 Trends in Biochemical Sci 2004 9 95 102 10.1016/j.tibs.2003.12.004 15102436

[B5] Webster MT Rozycka M Sara E Smalley M Young N Dale TC Wooster R Sequence variants of the axin gene in breast, colon and other cancers: an analysis of mutations that interfere with GSK-3 binding Genes Chromosomes Cancer 2000 28 443 453 10.1002/1098-2264(200008)28:4<443::AID-GCC10>3.0.CO;2-D 10862053

[B6] Cohen P Frame S The renaissance of GSK3 Nat Rev Mol Cell Biol 2001 2 769 776 11584304 10.1038/35096075

[B7] Jonak C Hirt H Glycogen synthase kinase 3/SHAGGY-like kinase in plants: an emerging family with novel functions Trends Plant Sci 2002 7 457 461 10.1016/S1360-1385(02)02331-2 12399181

[B8] Siegfried E Chou TB Perrimon N Wingless signaling acts through zeste-white 3 the *Drosophila* homolog of glycogen synthase kinase–3 to regulate engrailed and establish cell fate Cell 1992 71 1167 1179 10.1016/S0092-8674(05)80065-0 1335365

[B9] Heitzler P Simpson P The choice of cell fate in the epidermis of Drosophila Cell 1991 64 1083 1092 10.1016/0092-8674(91)90263-X 2004417

[B10] He X Saint-Jeannet JP Woodgett JR Varnus HE David IB Glycogen synthase kinase–3 and dorsoventral patterning in *Xenopus* embryos Nature 1995 374 617 622 10.1038/374617a0 7715701

[B11] Broun M Gee L Reinhardt B Bode H Formation of the head organizer in hydra involves the canonical Wnt pathway Development 2005 132 2907 2916 10.1242/dev.01848 15930119

[B12] Dornelas M Lejeune B Dron M Kreis M The *Arabidopsis* SHAGGY-related protein kinase (ASK) gene family: structure, organization and evolution Gene 1998 212 249 257 10.1016/S0378-1119(98)00147-4 9611268

[B13] Li J Nam KH Regulation of Brassinosteroid Signaling by a GSK3/SHAGGY-Like Kinase Science 2002 295 1299 1301 11847343 10.1126/science.1065769

[B14] Vert G Chory J Downstream nuclear events in brassinosteroid signalling Nature 2006 44 96 100 16672972 10.1038/nature04681

[B15] Perez-Perez JM Ponce MR Micol JL The UCU1 Arabidopsis gene encodes a SHAGGY/GSK3-like kinase required for cell expansion along the proximodistal axis Dev Biol 2002 242 161 173 10.1006/dbio.2001.0543 11820813

[B16] He J-X Gendron JM Yang Y Li J Wang Z-Y The GSK3-like BIN2 phosphorylates and destabilizes BZR1, a positive regulator of the brassinosteroid signaling pathway in Arabidopsis Proc Natl Acad Sci USA 2002 99 10185 10190 10.1073/pnas.152342599 12114546 PMC126645

[B17] Ryu H Kim K Cho H Park J Choe S Hwang I Nucleoplasmic shuttling of BZR1 mediated by phophorylation is essential in Arabidopsis brassinosteroid signalling Plant Cell 2007 19 2749 2762 10.1105/tpc.107.053728 17873094 PMC2048706

[B18] Ryu H Cho H Kim K Hwang I Phosphorylation dependent nucleoplasmic shuttling of BES1 is a key regulatory event in Brassinosteroid signalling Mol Cells 2010 29 283 290 10.1007/s10059-010-0035-x 20387034

[B19] Peng P Yan Z Zhu Y Li J Regulation of the Arabidopsis GSK3-like Kinase BRASSINOSTEROID-INSENSITIVE 2 through Proteasome-Mediated Protein Degradation Mol Plant 2008 1 338 346 10.1093/mp/ssn001 18726001 PMC2519614

[B20] Kim T-W Guan S Sun Y Deng Z Tang W Shang J-X Sun Y Burlingame AL Wang Z-Y Brassinosteroid signal transduction from cell-surface receptor kinases to nuclear transcription factors Nat Cell Biol 2009 11 1254 1262 10.1038/ncb1970 19734888 PMC2910619

[B21] Vert G Walcher CL Chory J Nemhauser JL Integration of auxin and brassinosteroid pathways by Auxin Response Factor 2 Proc Natl Acad Sci USA 2008 105 9829 9834 10.1073/pnas.0803996105 18599455 PMC2474533

[B22] Rozhon W Mayerhofer J Petutschnig E Fujioka S Jonak C ASKθ, a group-III Arabidopsis GSK3, functions in the brassinosteroid signalling pathway Plant J 2010 62 215 223 10.1111/j.1365-313X.2010.04145.x 20128883 PMC2881309

[B23] Dornelas MC Wittich P Von Recklinghausen I Van Lammeren A Kreis M Characterization of three novel members of the *Arabidopsis SHAGGY*-related protein kinases (ASK) multigene family Plant Mol Biol 1999 39 137 147 10.1023/A:1006102812280 10080716

[B24] Tavares R Vidal J van Lammeren A Kreis M AtSKθ, a plant homologue of SGG/GSK-3 marks developing tissues in *Arabidospsis thaliana* Plant Mol Biol 2002 50 261 271 10.1023/A:1016009831678 12175018

[B25] Dornelas MC Van Lammeren A Kreis M *Arabidopsis thaliana* SHAGGY-related protein kinases (AtSK11 and 12) function in perianth and gynoecium development Plant J 2000 21 419 429 10.1046/j.1365-313x.2000.00691.x 10758494

[B26] Kim TW Michniewicz M Bergmann DC Wang ZY Brassinosteroid regulates stomatal development by GSK3-mediated inhibition of a MAPK pathway Nature 2012 482 419 423 10.1038/nature10794 22307275 PMC3292258

[B27] Jonak C Beisteiner D Beyerly J Hirt H Wound-induced expression and activation of WIG, a novel glycogen synthase kinase 3 Plant Cell 2000 12 1467 1475 10948263 10.1105/tpc.12.8.1467 PMC149116

[B28] Diatchenko L Lau YFC Campbell AP Chenchik A Moqadam F Huang B Lukyanov S Lukyanov K Gurskaya N Sverdlov ED Siebert PD Suppression subtractive hybridization: A method for generating differentially regulated or tissue-specific cDNA probes and libraries Proc Natl Acad Sci USA 1996 93 6025 6030 10.1073/pnas.93.12.6025 8650213 PMC39182

[B29] Thompson JD Gibson TJ Plewniak F Jeanmougin F Higgins DG The ClustalX windows interface: flexible strategies for multiple sequence alignment aided by quality analysis tools Nucleic Acids Res 1997 25 4876 4882 10.1093/nar/25.24.4876 9396791 PMC147148

[B30] Howe K Bateman A Durbin R QuickTree: building huge Neighbour-Joining trees of protein sequences Bioinformatics 2002 18 1546 1547 10.1093/bioinformatics/18.11.1546 12424131

[B31] Edgar RC MUSCLE: multiple sequence alignment with high accuracy and high throughput Nucleic Acids Res 2004 32 1792 1797 10.1093/nar/gkh340 15034147 PMC390337

[B32] Katoh K Kuma K Toh H Miyata T MAFFT version 5: improvement in accuracy of multiple sequence alignment Nucleic Acids Res 2005 33 511 518 10.1093/nar/gki198 15661851 PMC548345

[B33] Sears ER Lewis KR, Riley R Nullisomic-tetrasomic combinations in hexaploid wheat Chromosome Manipulation and Plant Genetics 1966 Edinburgh, London: Oliver and Boyd 29 45

[B34] Hanks SK Quinn AM Protein kinase catalytic domain: sequence database Method Enzymol 1991 200 38 61 10.1016/0076-6879(91)00126-h 1956325

[B35] Dajani R Fraser E Roe SM Young N Good V Dale TC Pearl LH Crystal Structure of Glycogen Synthase Kinase 3β: Structural Basis for Phosphate-Primed Substrate Specificity and Autoinhibition Cell 2001 105 721 732 10.1016/S0092-8674(01)00374-9 11440715

[B36] ter Haar E Coll JT Austen DA Hsiao HM Swenson L Jain J Structure of GSK3-beta reveals a primed phosphorylation mechanism Nat Struct Biol 2001 8 593 596 10.1038/89624 11427888

[B37] Hughes K Nikolakaki E Plyte SE Totty NF Woodgett J Modulation of the glycogen synthase kinase 3-family by tyrosine phosphorylation EMBO J 1993 12 803 808 8382613 10.1002/j.1460-2075.1993.tb05715.x PMC413270

[B38] Doble BW Woodgett JR GSK-3: tricks of the trade for a multi-tasking kinase J Cell Sci 2003 116 1175 1186 10.1242/jcs.00384 12615961 PMC3006448

[B39] Peng P Zhao J Zhu Y Asami T Li J A direct docking mechanism for a plant GSK3-like kinase to phosphorylate its substrates J Biol Chem 2010 285 24646 24653 10.1074/jbc.M110.142547 20522560 PMC2915701

[B40] De Rybel B Audenaert D Vert G Rozhon W Mayerhofer J Peelman F Coutuer S Denayer T Jansen L Nguyen L Vanhoutte I Beemster GTS Vleminckx K Jonak C Chory J Inze D Russinova E Beeckman T Chemical inhibition of a subset of Arabidopsis thaliana GSK3-like kinases activates brassinosteroid signaling Chem Biol 2009 16 594 604 10.1016/j.chembiol.2009.04.008 19549598 PMC4854203

[B41] Yoo MJ Albert VA Soltis PS Soltis D Phylogenetic diversification of *glycogen synthase kinase 3/SHAGGY-like* kinase genes in plants BMC Plant Biol 2006 6 3 12 10.1186/1471-2229-6-3 16504046 PMC1524769

[B42] The International Brachypodium Initiative Genome sequencing and analysis of the model grass Brachypodium distachyon Nature 2010 463 763 768 10.1038/nature08747 20148030

[B43] Bowers JE Chapman BA Rong J Paterson AH Unravelling angiosperm genome evolution by phylogenetic analysis of chromosomal duplication events Nature 2003 422 433 438 10.1038/nature01521 12660784

[B44] Henry Y Bedhomme M Blanc G History, protohistory and prehistory of the *Arabidopsis thaliana* chromosome complement Trends Plant Sci 2006 11 267 273 10.1016/j.tplants.2006.04.002 16690345

[B45] Jiao Y Wickett NJ Ayyampalayam S Chanderbali AS Landherr L Ralph PE Tomsho LP Hu Y Liang H Soltis PS Soltis DE Clifton SW Schlarbaum SE Schuster SC Ma H Leebens-Mack J DePamphilis CW Ancestral polyploidy in seed plants and angiosperms Nature 2011 473 97 100 10.1038/nature09916 21478875

[B46] The French–Italian Public Consortium for Grapevine Genome Characterization The grapevine genome sequence suggests ancestral hexaploidization in major angiosperm phyla Nature 2007 449 463 467 10.1038/nature06148 17721507

[B47] Ming R The draft genome of the transgenic tropical fruit tree papaya (Carica papaya Linnaeus) Nature 2008 452 991 996 10.1038/nature06856 18432245 PMC2836516

[B48] Barker M Vogel H Schranz ME Paleopolyploidy in the Brassicales: Analyses of the Cleome Transcriptome Elucidate the History of Genome Duplications in Arabidopsis and Other Brassicales Genome Biol Evol 2009 1 391 399 20333207 10.1093/gbe/evp040 PMC2817432

[B49] Gardiner J Schroeder S Polacco ML Sanchez-Villeda H Fang Z Morgante M Landewe T Fengler K Useche F Hanafey M Tingey S Chou H Wing R Soderlund C Coe EH Jr Anchoring 9,371 Maize Expressed Sequence Tagged Unigenes to the Bacterial Artificial Chromosome Contig Map by Two-Dimensional Overgo Hybridization Plant Physiol 2004 134 1317 1326 10.1104/pp.103.034538 15020742 PMC419808

[B50] Alexandrov NN Brover VV Freidin S Troukhan ME Tatarinova TV Zhang H Swaller TJ Lu Y-P Bouck J Flavell RB Feldmann KA Insights into corn genes derived from large-scale cDNA sequencing Plant Mol Biol 2009 69 179 194 10.1007/s11103-008-9415-4 18937034 PMC2709227

[B51] Yan Z Zhao J Peng P Chihara RK Li J BIN2 Functions Redundantly with Other Arabidopsis GSK3-Like Kinases to Regulate Brassinosteroid Signaling Plant Physiol 2009 150 710 721 10.1104/pp.109.138099 19395409 PMC2689954

[B52] Rensing SA Ick J Fawcett JA Lang D Zimmer A Van de Peer Y Reski R An ancient genome duplication contributed to the abundance of metabolic genes in the moss *Physcomitrella patens* BMC Evol Biol 2007 7 130 10.1186/1471-2148-7-130 17683536 PMC1952061

[B53] Chen GP Ma WS Huang ZJ Xu T Xue XB Shen YZ Isolation and characterization of TaGSK1 involved in wheat salt tolerance Plant Science 2003 165 1369 1375 10.1016/S0168-9452(03)00365-0

[B54] Yin Y Wang ZY Mora-Garcia S Li J Yoshida S Asami T Chory J BES1 accumulates in the nucleus in response to brassinosteroids to regulate gene expression and promote stem elongation Cell 2002 109 181 191 10.1016/S0092-8674(02)00721-3 12007405

[B55] Feldman M Levy AA Allopolyploidy - a shaping force in the evolution of wheat genomes Cytogenet Genome Res 2005 109 250 258 10.1159/000082407 15753584

[B56] Adams KL Cronn R Percifield R Wendel JF Genes duplicated by polyploidy show unequal contributions to the transcriptome and organ-specific reciprocal silencing Proc Natl Acad Sci USA 2003 100 4649 4654 10.1073/pnas.0630618100 12665616 PMC153610

[B57] Flagel L Udall J Nettleton D Wendel J Duplicate gene expression in allopolyploid *Gossypium* reveals two temporally distinct phases of expression evolution BMC Biol 2008 6 16 10.1186/1741-7007-6-16 18416842 PMC2330141

[B58] Woodgett JR Molecular cloning and expression of glycogen synthase kinase-3/factor A EMBO J 1990 9 2431 2438 2164470 10.1002/j.1460-2075.1990.tb07419.x PMC552268

[B59] Mukai F Ishiguro K Sano Y Fujita SC Alternative splicing isoform of tau protein kinase I/glycogen synthase kinase-3beta J Neurochem 2002 81 1073 1083 10.1046/j.1471-4159.2002.00918.x 12065620

[B60] Bourouis M Moore P Ruel L Grau Y Heitzler P Simpson P An early embryonic product of the gene shaggy encodes a serine/threonine protein kinase related to the CDC28/cdc2+ subfamily EMBO J 1990 9 2877 2884 2118107 10.1002/j.1460-2075.1990.tb07477.x PMC552001

[B61] Theißen G Kim JT Saedler H Classification and Phylogeny of the MADS Box Multigene family suggest defined roles of MADS Box gene subfamilies in the morphological evolution of Eukaryotes J Mol Evol 1996 43 484 516 10.1007/BF02337521 8875863

[B62] Becker A Theißen G The major clades of MADS-box genes and their role in the development and evolution of flowering plants Mol Phylogenet Evol 2003 29 464 489 10.1016/S1055-7903(03)00207-0 14615187

[B63] De Bodt S Maere S Van de Peer Y Genome duplication and the origin of angiosperms Trends in Ecology and Evol 2005 20 591 597 10.1016/j.tree.2005.07.008 16701441

[B64] Jiao Y Leebens-Mack J Ayyampalayam S Bowers JE McKain MR McNeal J Rolf M Ruzicka DR Wafula E Wickett NJ Wu X Zhang Y Wang J Carpenter EJ Deyholos MK Kutchan TM Chanderbali AS Soltis PS Stevenson DW McCombie R Pires CJ Wong GK Soltis DE Depamphilis CW A genome triplication associated with early diversification of the core eudicots Genome Biol 2012 13 R3 10.1186/gb-2012-13-1-r3 22280555 PMC3334584

[B65] Tang H Bowers JE Wang X Paterson AH Angiosperm genome comparisons reveal early polyploidy in the monocot lineage Proc Natl Acad Sci USA 2010 107 472 477 10.1073/pnas.0908007107 19966307 PMC2806719

[B66] Seoighe C Gehring C Genome duplication led to highly selective expansion of the *Arabidopsis thaliana* proteome Trends in Genet 2004 20 461 464 10.1016/j.tig.2004.07.008 15363896

[B67] Van de Peer Y Maere S Meyer A The evolutionary significance of ancient genome duplications Nat Rev Genet 2009 10 725 732 10.1038/nrg2600 19652647

[B68] Lang D Weiche B Timmerhaus G Richardt S Riaño-Pachón DM Corrêa LGG Reski R Mueller-Roeber B Rensing SA Genome-Wide Phylogenetic Comparative Analysis of Plant Transcriptional Regulation: A Timeline of Loss, Gain, Expansion, and Correlation with Complexity *Genome* Biol Evol 2010 2 488 503 10.1093/gbe/evq032 PMC2997552 20644220

[B69] Campagne S Auxin-mediated embryonic pattern formation in wheat (T. aestivum). PhD thesis 2005 Freiburg University: Faculty of Biology, Cell Biology Department

[B70] Liu YG Mitsukawa N Oosumi T Whittier RF Efficient isolation and mapping of Arabidopsis thaliana T-DNA insert junctions by thermal asymmetric interlaced PCR Plant J 1995 8 457 463 10.1046/j.1365-313X.1995.08030457.x 7550382

[B71] Liu YG Chen Y High-efficiency thermal asymmetric interlaced PCR for amplification of unknown flanking sequences Biotechniques 2007 43 649 650 652, 654 passim 10.2144/000112601 18072594

[B72] Ausubel FM, Brent R, Kingston RE, Moore DD, Seidmann JG, Smith JA, Struhl E Current protocols in molecular biology Volume 3 2003 New York: John Wiley and Sons

[B73] Clamp M Cuff J Searle SM Barton GJ The Jalview Java alignment editor Bioinformatics 2004 20 426 427 10.1093/bioinformatics/btg430 14960472

[B74] Frickenhaus S Beszteri B Quicktree-SD: Software developed by AWI-Bioinformatics 2008 http://hdl.handle.net/10013/epic.33164

[B75] Schmidt HA Strimmer K Vingron M von Haeseler A TREE-PUZZLE: maximum likelihood phylogenetic analysis using quartets and parallel computing Bioinformatics 2002 18 502 504 10.1093/bioinformatics/18.3.502 11934758

[B76] Abascal F Zardoya R Posada D ProtTest: selection of best-fit models of protein evolution Bioinformatics 2005 21 2104 2105 10.1093/bioinformatics/bti263 15647292

