# Supplementary material for: Identification and characterization of two wheat Glycogen Synthase Kinase 3/ SHAGGY-like kinases
Source: BMC Plant Biol. 2013 Apr 18;13:64. doi: 10.1186/1471-2229-13-64 (PMC3637598; doi:10.1186/1471-2229-13-64)
Supplement: Additional file 1 — List of plant GSKs included in the phylogenic analysis. [file 1471-2229-13-64-S1.docx]

**Additional File 1: List of plant GSKs included in the phylogenic analysis**

| Species | Gene Name | Locus | Acc. Protein |  | Databases |
| --- | --- | --- | --- | --- | --- |
| *Arabidopsis thaliana* | ASKalpha-ATSK11 | AT5G26751 |  |  | TAIR |
|  | ASKbeta-ASK2 | AT3G61160 |  |  |  |
|  | ASKgamma-ATSK12 | AT3G05840 |  |  |  |
|  | ASKdelta-ATSK42 | AT1G57870 |  |  |  |
|  | ASKepsilon-ATSK13 | AT5G14640 |  |  |  |
|  | ASKdzeta-ATSK23 | AT2G30980 |  |  |  |
|  | ASKetha-ATSK21-BIN2 | AT4G18710 |  |  |  |
|  | ASKtetha-ATSK32 | AT4G00720 |  |  |  |
|  | ASKkappa-ATSK41 | AT1G09840 |  |  |  |
|  | ASKiota-ATSK22 | AT1G06390 |  |  |  |
| *Oryza sativa* | OsGSK1 | LOC_Os01g14860 |  |  | RGAP |
|  | OsGSK2-OSKdzeta | LOC_Os01g10840 |  |  |  |
|  | OsGSK3 | LOC_Os01g19150 |  |  |  |
|  | OsGSK4 | LOC_Os02g14130 |  |  |  |
|  | OsGSK5 | LOC_Os03g62500 |  |  |  |
|  | OsGSK6 | LOC_Os05g04340 |  |  |  |
|  | OsGSK7 | LOC_Os05g11730 |  |  |  |
|  | OsGSK8-OSKetha | LOC_Os06g35530 |  |  |  |
|  | OsGSK9 | LOC_Os10g37740 |  |  |  |
| *Brachypodium dist.* |  |  | BRADI2G06490.1 |  | EnsemblPlants |
|  |  |  | BRADI2G32620.3 |  |  |
|  |  |  | BRADI3G09067.1 |  |  |
|  |  |  | BRADI2G08890.1 |  |  |
|  |  |  | BRADI2G38590.1 |  |  |
|  |  |  | BRADI2G11370.1 |  |  |
|  |  |  | BRADI1G02160.1 |  |  |
|  |  |  | BRADI3G31480.1 |  |  |
| *Triticum aestivum* |  |  | AK330872 |  | GenBank |
|  |  |  | AK332715 |  |  |
|  | TaGSK1 |  | AAM77397 |  |  |
|  | TaSK5 |  | BAF36565 |  |  |
| *Hordeum vulgare* |  |  | BAJ89558 |  | GenBank |
|  |  |  | BAJ96026 |  |  |
|  |  |  | BAJ99594 |  |  |
|  |  |  | AK251287 |  |  |
|  |  |  | BAJ93751 |  |  |
| *Zea mays* |  |  | **ACG34459** | A | GenBank |
|  |  |  | ACF85585 |  | GenBank |
|  |  |  | AY104068 |  | GenBank |
|  |  |  | GRMZM2G472625_P01 |  | EnsemblPlants |
|  |  |  | **NP_001149135** | B | GenBank |
|  |  |  | ACN32041 |  | GenBank |
|  |  |  | ACG33866 |  | GenBank |
|  |  |  | GRMZM2G045330_P01 |  | EnsemblPlants |
|  |  |  | **ACG33869** | C | GenBank |
|  |  |  | NP_001130511 |  | GenBank |
|  |  |  | AY106255 |  | GenBank |
|  |  |  | NP_001149983 |  | GenBank |
|  |  |  | GRMZM2G121790_P01 |  | EnsemblPlants |
|  |  |  | **ACN27945** | D | GenBank |
|  |  |  | NP_001167685 |  | GenBank |
|  |  |  | GRMZM2G043350_P01 |  | EnsemblPlants |
|  |  |  | **AY103545** | E | GenBank |
|  |  |  | NP_001150105 |  | GenBank |
|  |  |  | NP_001148880 |  | GenBank |
|  |  |  | **ACF80579** | F | GenBank |
|  |  |  | GRMZM2G155836_P01 |  | EnsemblPlants |
|  |  |  | **ACF79716** | G | GenBank |
|  |  |  | ACN34914 |  | GenBank |
|  |  |  | NP_001105536 |  | GenBank |
|  |  |  | GRMZM2G138676_P01 |  | EnsemblPlants |
|  |  |  | **GRMZM2G024151_P01** | H | EnsemblPlants |
|  |  |  | **GRMZM2G109624_P01** | I | EnsemblPlants |
|  |  |  | **GRMZM2G131853_P01** | J | EnsemblPlants |
| *Physcomitrella patens* |  |  | Pp1S306_43V6.1 |  | Phytozome |
|  |  |  | Pp1S306_62V6.1 |  |  |
|  |  |  | Pp1S60_100V6.1 |  |  |
|  |  |  | Pp1S60_85V6.1 |  |  |
|  |  |  | Pp1S52_55V6.1 |  |  |
|  |  |  | Pp1S74_168V6.1 |  |  |
|  |  |  | Pp1S391_47V6.1 |  |  |
| *Aquilegia coerulea* |  |  | Aquca_013_00389.1 |  | Phytozome |
|  |  |  | Aquca_039_00097.1 |  |  |
|  |  |  | Aquca_003_00407.2 |  |  |
|  |  |  | Aquca_025_00356.1 |  |  |
|  |  |  | Aquca_021_00184.1 |  |  |
| *Carica papaya* |  |  | CARPA_733.2 |  | ASGPB |
|  |  |  | CARPA_52.151 |  |  |
|  |  |  | CARPA_48.204 |  |  |
|  |  |  | CARPA_386.4 |  |  |
|  |  |  | CARPA_113.29 |  |  |
| *Vitis vinifera* |  |  | Vv14s0066g01050.t01 |  | EnsemBlplants |
|  |  |  | Vv14s0060g00600.t01 |  |  |
|  |  |  | Vv07s0005g02100.t01 |  |  |
|  |  |  | Vv15s0048g02010.t01 |  |  |
|  |  |  | Vv12s0035g01060.t01 |  |  |
|  |  |  | Vv10s0003g01480.t01 |  |  |
|  |  |  | Vv12s0028g01810.t01 |  |  |

### Protein or cDNA accessions of *Triticum aestivum* and *Hordeum vulgare* were obtained from Plant GDB and GenBank databases. For *Zea mays*, 28 proteins were identified in the Genbank and EnsemblPlants databases. Within these 28 sequences, 10 subgroups of proteins (A-J) whose members shared high identity (97 to 99%) were identified. For each subgroup, one member was selected as representative for this subgroup and was highlighted in bold. Protein accessions of *Physcomitrella patens* originated from the EnsemblPlants and Phytozome databases. An *Arabidopsis* cyclin dependant kinase [TAIR: AT1G73690.1] and an *Arabidopsis* protein kinase both not belonging to the GSKs [TAIR: AT1G67580.1] were selected as outgroup sequences. RGAP: Rice genome annotation project databases; ASGPB: Hawaii Papaya Genome Project ASGPB.
