# Supplementary figures and images for: Identification and characterization of two wheat Glycogen Synthase Kinase 3/ SHAGGY-like kinases
Source: BMC Plant Biol. 2013 Apr 18;13:64. doi: 10.1186/1471-2229-13-64 (PMC3637598; doi:10.1186/1471-2229-13-64)

Additional File 2: Neighbour-Joining Tree

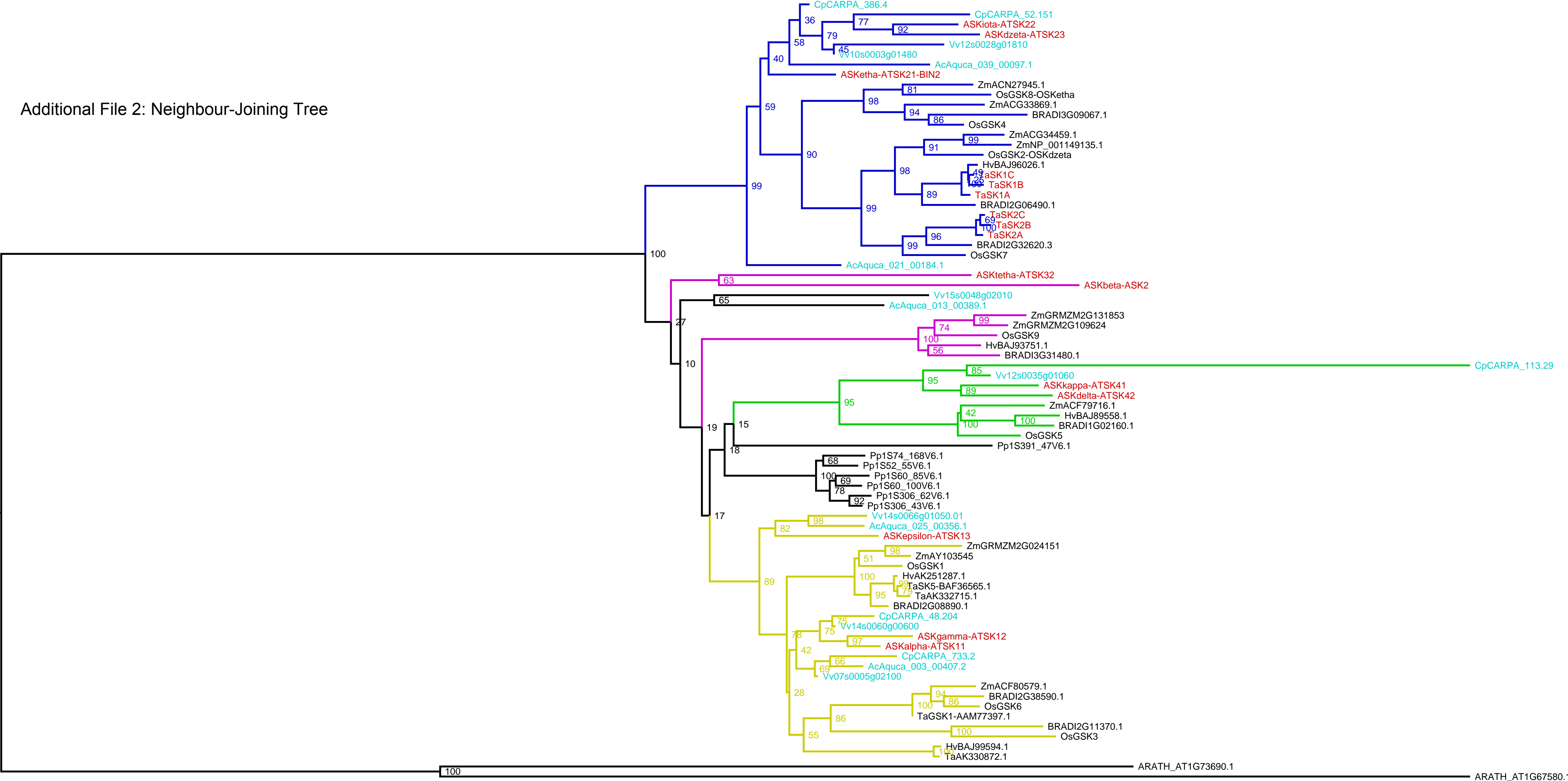

Supplement: Additional file 2 — Neighbour-Joining tree. [file 1471-2229-13-64-S2.pdf]

98

ARATH\_O2

ARATH\_O1

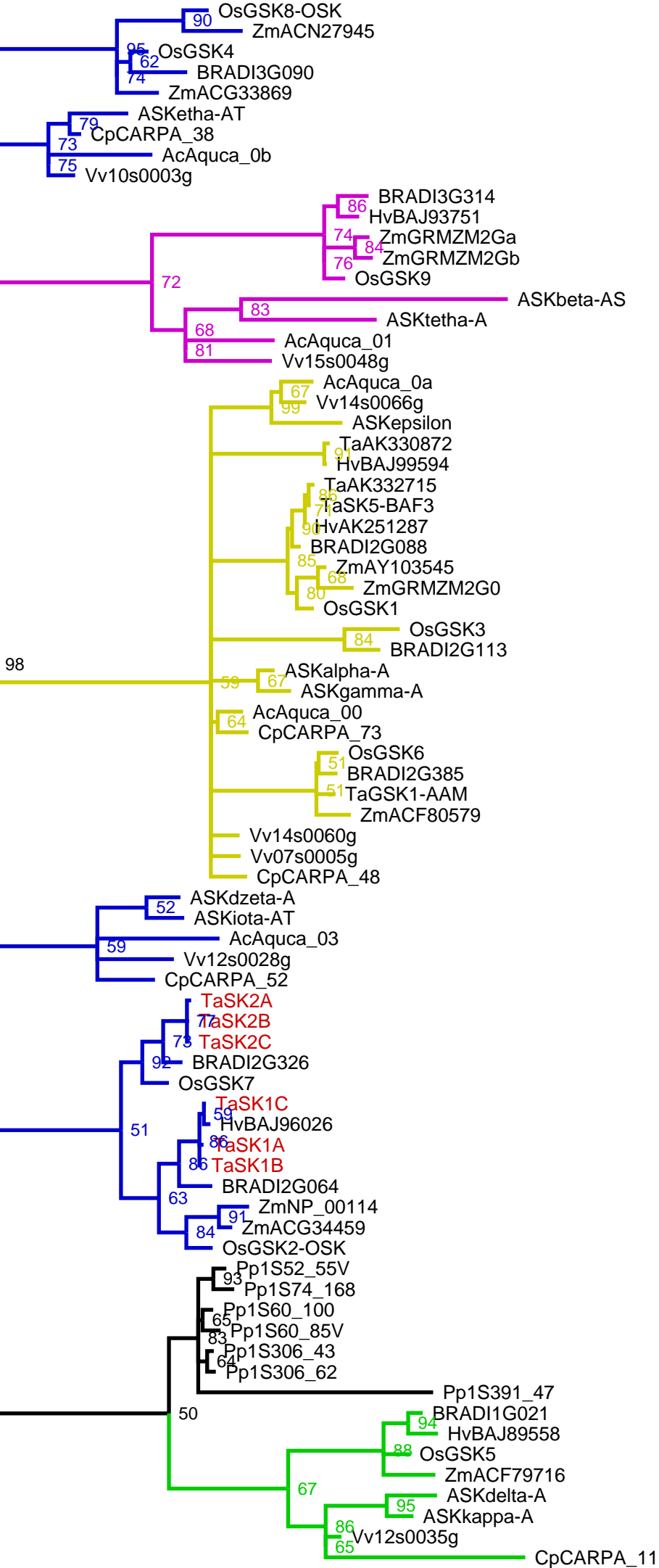

0 . 6

Additional File 3: Maximum Likelihood Tree

Supplement: Additional file 3 — Maximum Likelihood (ML) tree. [file 1471-2229-13-64-S3.pdf]
